# British Society for Rheumatology guideline on prescribing drugs in pregnancy and breastfeeding: comorbidity medications used in rheumatology practice

**DOI:** 10.1093/rheumatology/keac552

**Published:** 2022-11-02

**Authors:** Karen Schreiber, Margreta Frishman, Mark D Russell, Mrinalini Dey, Julia Flint, Alexander Allen, Amy Crossley, Mary Gayed, Kenneth Hodson, Munther Khamashta, Louise Moore, Sonia Panchal, Madeleine Piper, Clare Reid, Katherine Saxby, Naz Senvar, Sofia Tosounidou, Maud van de Venne, Louise Warburton, David Williams, Chee-Seng Yee, Caroline Gordon, Ian Giles, BSR Standards, Audit and Guidelines Working Group, Ian Giles, Ed Roddy, Kate Armon, Lauren Astell, Caroline Cotton, Alan Davidson, Sarah Fordham, Claire Jones, Christopher Joyce, Anoop Kuttikat, Zoe McLaren, Karen Merrison, Devesh Mewar, Amanda Mootoo, Emma Williams

**Affiliations:** Thrombosis & Haemophilia Centre, Guy's and St Thomas' NHS Foundation Trust, London, UK; Department of Rheumatology, Danish Hospital for Rheumatic Diseases, Sonderborg, Denmark; Department of Regional Health Research, University of Southern Denmark, Odense, Denmark; Obstetrics and Gynaecology, North Middlesex University Hospital NHS Trust, London, UK; Centre for Rheumatic Diseases, King’s College London, London, UK; Institute of Life Course and Medical Sciences, University of Liverpool, Liverpool, UK; Department of Rheumatology, Robert Jones and Agnes Hunt Orthopaedic Hospital NHS Foundation Trust, Shropshire, UK; Clinical Affairs, British Society for Rheumatology, London, UK; Patient Representative, London, UK; Rheumatology, University Hospital Birmingham NHS Foundation Trust, Birmingham, UK; The UK Teratology Information Service, Newcastle upon Tyne, UK; Division of Women’s Health, Lupus Research Unit, King's College London, London, UK; Rheumatic and Musculoskeletal Disease Unit, Our Lady’s Hospice and Care Service, Dublin, Ireland; Rheumatology, South Warwickshire NHS Foundation Trust, Warwickshire, UK; Royal National Hospital for Rheumatic Diseases, Royal United Hospital, Bath, UK; Patient Representative, London, UK; Pharmacology, University College London Hospitals NHS Foundation Trust, London, UK; Obstetrics and Gynaecology, St George's University Hospitals NHS Foundation Trust, London, UK; Lupus UK Centre of Excellence, Sandwell and West Birmingham NHS Trust, Birmingham, UK; Women’s Health, Frimley Park Hospital, Surrey, UK; Shropshire Community NHS Trust, Shropshire, UK; Primary Care and Health Sciences, Keele University, Keele, UK; Obstetrics, University College London Hospitals NHS Foundation Trust, London, UK; Department of Rheumatology, Doncaster and Bassetlaw, Teaching Hospitals NHS Foundation Trust, Doncaster, UK; Rheumatology Research Group, Institute of Inflammation and Ageing, University of Birmingham, Birmingham, UK; Centre for Rheumatology, Department of Inflammation, Division of Medicine, University College London, London, UK

**Keywords:** rheumatic disease, pregnancy, breastfeeding, prescribing, analgesics, NSAIDs, anticoagulants, antihypertensive drugs


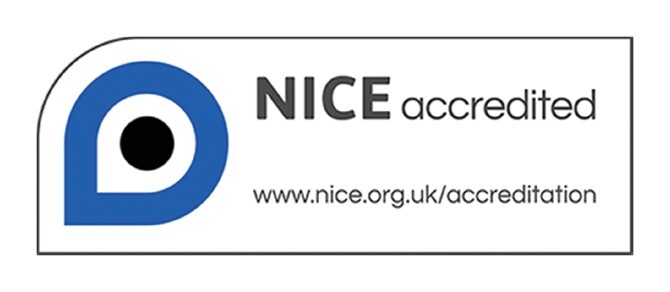


NICE has accredited the process used by BSR to create its clinical guidelines. The term began on 27 February 2012 and the current renewed accreditation is valid until 31 December 2023. More information on accreditation can be viewed at www.nice.org.uk/accreditation.

## Scope and purpose

### Background

The rationale behind this update on the 2016 British Society for Rheumatology (BSR) guidelines on prescribing anti-rheumatic drugs in pregnancy and breastfeeding [1, 2] was described in detail in the guideline scope [3]. In brief, despite the existence of additional evidence-based guidelines on prescribing/managing rheumatic disease in pregnancy [4–7] the information contained within them requires continual review to include emerging information on the safety of new and existing drugs in pregnancy.

Chronic disease adversely affects pregnancy. Data from Mothers and Babies: Reducing Risk through Audits and Confidential Enquiries across the UK (MBRRACE-UK), reports regularly from a national programme of work conducting surveillance and investigating the causes of maternal deaths, stillbirths and infant deaths [8]. Data from 2017–19 found that 8.8 women per 100 000 died during pregnancy or up to six weeks after childbirth or the end of pregnancy, and most women who died had multiple health problems or other vulnerabilities [8]. In all decisions regarding medication choices and changes, it is also important to consider the potential for deterioration in the mother's wellbeing through side effects or reduced disease control (and its adverse impact on the baby). Therefore, the exposure of the foetus to different drugs when switches are made must be balanced against possible foetal gains and understanding the potential impact of reduced control of the medical disorder on a pregnancy is vital [9].

### Need for guideline

Patients with inflammatory rheumatic disease (IRD) should be counselled to achieve and then maintain remission or low disease activity before/during pregnancy to reduce the risk of adverse pregnancy outcomes [10]. This goal is primarily achieved through adjustment of therapy to ensure disease control with disease modifying anti-rheumatic drugs (DMARDs) and/or immunosuppressive drugs that are compatible with pregnancy. These medications are reviewed in the BSR guideline on prescribing drugs in pregnancy and breastfeeding: immunomodulatory anti-rheumatic drugs and corticosteroids [11]. Many patients with IRD, however, have an additional burden of pain and comorbid illness [12] that require treatment with other medications. The compatibility of various comorbidity medications relevant to rheumatic disease will be covered in this update. This updated information will provide advice for healthcare professionals and patients to ensure more confident prescribing in these scenarios and will highlight any medications that should be stopped and/or avoided in the reproductive age group unless highly effective contraception is used, in line with guidance issued by the Medicines and Healthcare Products Regulatory Agency (MHRA) and Faculty of Sexual and Reproductive Healthcare [13, 14].

### Objectives of guideline

To update the previous BSR guidelines on prescribing in pregnancy in rheumatic disease for the following drug categories: pain management; NSAIDs and low-dose aspirin (LDA); anticoagulants; colchicine; dapsone; bisphosphonates; anti-hypertensives; and pulmonary vasodilators. This revised guideline was produced by consensus review of current evidence to answer specific questions in relation to each drug as follows. Should it be stopped pre-conception? Is it compatible with pregnancy? Is it compatible with breastmilk exposure? Where possible, recommendations are made regarding compatibility with paternal exposure.

### Target audience

The primary audience consists of health professionals in the UK directly involved in managing patients with rheumatic disease who are (or are planning to become) pregnant and/or breastfeeding, men planning to conceive, and patients who have unintentionally conceived while taking these medications. This audience includes rheumatologists, rheumatology nurses/allied health professionals, rheumatology speciality trainees and pharmacists, as well as the patients themselves. The guideline will also be useful to obstetricians, obstetric physicians, renal physicians, dermatologists and general practitioners who may prescribe these medications to patients in pregnancy.

This guideline uses the terms ‘woman’, ‘maternal’ or ‘mother’ throughout. These should be taken to include people who do not identify as women but are pregnant or have given birth [15]. Where the term ‘breastfeeding’ is used in this guideline it also refers to infant breastmilk exposure via other methods (e.g. expressed breastmilk, administered via a bottle).

### The areas the guideline does not cover

This guideline does not cover the management of infertility or acute pain relief during labour, hence morphine was excluded. Other drug categories: antimalarials; corticosteroids; disease modifying anti-rheumatic and immunosuppressive therapies; and biologic drugs are considered in another guideline [11]. All recommendations in this guideline were formulated by the working group on the basis of published evidence at the time of the systematic literature search, and do not necessarily refer to licensing information or Summary of Product Characteristics for individual medications.

### Stakeholder involvement

This guideline was commissioned by the BSR Standards, Audit and Guidelines Working Group. A Guideline Working group (GWG) was created, consisting of a chair (I.G.), alongside representatives from relevant stakeholders (Table 1). In accordance with BSR policy, all members of the GWG made declarations of interest, available on the BSR website.

**Table 1.** Composition of Guideline Working Group: list of group members and relevant stakeholders

Tasks	Role	PICO definition	Data search	Data extraction	Voting member	Non-voting member	Manuscript authors
Karen Schreiber	Lead author, trainee rheumatologist	x	x	x	x		x
Margreta Frishman	Lead author, trainee obstetrician	x	x	x	x		x
Mark Russell	Trainee rheumatologist	x			x		
Mrinalini Dey	Trainee rheumatologist	x			x		
Julia Flint	Trainee rheumatologist	x			x		
Alexander Allen	Data analyst	x	x			x	
Amy Crossley	Patient representative					x	
Mary Gayed	Consultant rheumatologist	x			x		
Kenneth Hodson	Head of UK Tetralogy Information Service & consultant obstetrician	x			x		
Munther Khamashta	Consultant rheumatologist	x			x		
Louise Moore	Clinical nurse specialist	x			x		
Sonia Panchal	Consultant rheumatologist	x			x		
Madeleine Piper	Consultant rheumatologist	x			x		
Clare Reid	Patient representative	x				x	
Katherine Saxby	Pharmacist	x			x		
Naz Senvar	Trainee obstetrician	x			x		
Sofia Tosounidou	Consultant rheumatologist	x			x		
Maud van de Venne	Consultant obstetrician	x			x		
Louise Warburton	General practitioner	x			x		
David Williams	Consultant obstetric physician	x			x		
Chee-Seng Yee	Consultant rheumatologist	x			x		
Caroline Gordon	Consultant rheumatologist	x			x		
Ian Giles	Chair of working group & consultant rheumatologist	x	x	x	x		x

All members were involved in data review, formulation of recommendations and editing of the manuscript.

### Involvement and affiliations of stakeholder groups involved in guideline development

The GWG consisted of rheumatologists from a range of clinical backgrounds, various allied health professionals, other specialists in women’s health, lay members and representatives from the United Kingdom Tetralogy Information Service (UKTIS). All members of the working group contributed to the process for agreeing key questions, guideline content, recommendations and strength of agreement.

## Rigour of development

### Statement of scope of literature search and strategy employed

Most medications covered in this guideline have been comprehensively and systematically reviewed in multiple other documents, since the first BSR guideline on this topic. Therefore, a consensus-based approach was taken to compile and assess most significant evidence published since 2013 to December 2020 through a comprehensive search of MEDLINE, PubMed and EMBASE databases with specific search terms (Supplementary Table S1, available at *Rheumatology* online). Filters were applied to capture National Institute for Health and Care Excellence (NICE) guidance, international guidelines, systematic reviews, cohort studies or case series. Information was preferentially selected from NICE guidance and/or largest/most recent systematic reviews and where lacking was extracted from largest cohort, case series or abstract. Findings were cross-referenced with the previous BSR guideline [2], as well as the Cochrane, Lactmed (a National Library of Medicine database on drugs and lactation) and UKTIS databases.

**Figure 1.**
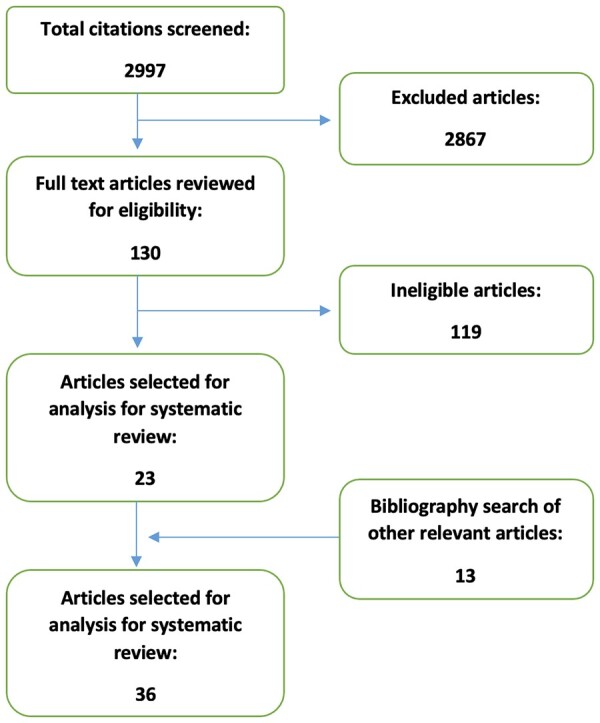
Flow diagram of study selection

Two independent reviewers screened the title and abstract of 2997 articles, identified 130 and selected the most recent/largest systematic reviews or largest cohort study or case series as well as any NICE guidance and international guidelines. Thirty-six studies (Fig. 1) met the inclusion criteria and relevant information was extracted into data-extraction tables.

### Statement of methods used to formulate the recommendations (levels of evidence)

The working group met regularly to formalise search strategy, review evidence, resolve disagreements and finally to determine recommendations. This guideline was developed in line with BSR’s Guidelines Protocol using Grading of Recommendations, Assessment, Development and Evaluations (GRADE) methodology to determine quality of evidence and strength of recommendation. Accompanying each recommendation in this guideline, in brackets, is the strength of recommendation, quality of evidence and strength of agreement (SOA).

### Strength of recommendation

Using GRADE, recommendations were categorized as either strong (denoted by 1) or weak (denoted by 2), according to the balance between benefits and risks. A strong recommendation was made when the benefits clearly outweigh the risks (or vice versa). A weak recommendation denotes that the benefits are more closely balanced with the risk, or more uncertain.

### Quality of evidence

Using the GRADE approach, the quality of evidence was determined as either high (A), moderate (B) or low/very low (C) reflecting the confidence in the estimates of benefits or harm.

### Strength of agreement

The wording of each recommendation was discussed until all members were satisfied they would score at least 80 on a scale of 1 (no agreement) to 100 (complete agreement) and then 20/23 members with full voting rights scored each recommendation on the same scale and the average was calculated to generate a strength of agreement (SOA) score. Two patient representatives and a data-analyst expressed concern that they did not have sufficient medical knowledge of all drugs reviewed to score all recommendations, so while they fully agreed with each, they did not wish to score each one and did not contribute to the final SOA score.

### Statement of any limits of search and when guideline will be updated

The search was conducted in December 2020. Limits were placed for English language and filters as described above. The guideline will be updated in five years.

## The guideline

Drugs are considered in the following categories: pain management; NSAIDs and LDA in the management of multisystem rheumatic disease; anticoagulants; bisphosphonates; anti-hypertensive medication in the management of multisystem rheumatic disease; and pulmonary vasodilators. The overall findings for maternal and foetal breastmilk exposures to each drug, including information and key references from the previous BSR guideline [2] are summarised and recommendations updated accordingly. Paternal exposures and recommendations are described separately after maternal data. An overall summary of compatibility of each drug pre-conception, during pregnancy, breastmilk exposure and paternal exposure is shown in Table 2. Generic recommendations were developed based on evidence as shown in Supplementary Table S2, available at *Rheumatology* online.

**Table 2.** Summary of drug compatibility in pregnancy and breastfeeding

	Compatible peri-conception	Compatible with 1st trimester	Compatible with 2nd/3rd trimester	Compatible with breastfeeding	Compatible with paternal exposure
**Conventional painkillers**					
Paracetamol	Yes	Yes^a^	Yes^a^	Yes	Yes^b^
Codeine	Yes	Yes	Yes	Yes^a^	Yes^b^
Tramadol	No	No	Yes^a^	Yes^c^	Yes^b^
**Other chronic pain treatments**					
Amitriptyline	Yes	Yes	Yes	Yes	Yes^b^
Gabapentin	Yes	Yes^d^	Yes^d^	Yes	Yes^b^
Pregabalin	Yes	Yes^d^	Yes^d^	Yes	Yes^b^
Venlafaxine	Yes	Yes	Yes	Yes^e^	Yes^b^
Fluoxetine	Yes	Yes	Yes	Yes^c^,e	Yes^b^
Paroxetine	Yes	Yes	Yes	Yes^c^^,^^e^	Yes^b^
Sertraline	Yes	Yes	Yes	Yes^c^^,^^e^	Yes^b^
Duloxetine	Yes	Yes	Yes	Yes^e^	Yes^b^
**NSAIDS**					
NSAIDs	Yes	Yes^a^^,^^f^	Stop by week 30	Yes	Yes
COX-2 inhibitors	No	No	No	No	Yes^b^
**Other drugs**					
Colchicine	Yes	Yes	Yes	Yes	Yes^b^
Dapsone	Yes	Yes	Yes	Yes	Yes^b^
**Anti-platelet agents**					
LDA	Yes	Yes	Yes	Yes	Yes^b^
Clopidogrel	Yes^c^	Yes^c^	Yes^c^	Yes^c^	Yes^b^
**Anticoagulants**					
Warfarin	No	No	Exceptional circumstances only	Yes	Yes^b^
LMWH	Yes	Yes	Yes	Yes	Yes^b^
DOACs	No	No	No	Rivaroxaban only	Yes^b^
Fondaparinux	Yes^c^	Yes	Yes	Yes	Yes^b^
**Bisphosphonates**					
Bisphosphonates	Stop 3 months in advance	No	No	No data	Yes^b^
**Antihypertensives**					
ACEi/ARBs	Stop when pregnancy confirmed		Exceptional circumstances only	Yes (enalapril)^c^	Yes^b^
Nifedipine	Yes	Yes <90 mg/day	Yes <90 mg/day	Yes	Yes^b^
Amlodipine	Yes^c^	Yes^c^	Yes^c^	Yes^c^	Yes^b^
Labetalol*	Yes	Yes	Yes	Yes	Yes^b^
Methyldopa*	Yes	Yes	Yes	Yes	Yes^b^
**Pulmonary vasodilators**					
Sildenafil	Multi-disciplinary team assessment			No data	Yes^b^
Bosentan	Multi-disciplinary team assessment			No data	Yes^b^
Prostacyclines	Multi-disciplinary team assessment			No data	Yes^b^

For further information and caveats, see relevant recommendations and main text in Executive Summary and full Guideline.

a Intermittent use advised – see main text for details.

b Based on limited data and no association with adverse foetal development or pregnancy outcome; therefore, unlikely to be harmful.

c Limited evidence, but unlikely to be harmful.

d Limited evidence regarding use for treatment of chronic pain in pregnancy. High-dose folic acid (5 mg/day) recommended.

e Cessation of anti-depressant therapy in post-natal period is not recommended.

f Possible association with miscarriage and malformation.

* Drugs not included in original search, but added due to relevance.

### Generic recommendations on prescribing in rheumatic disease in pregnancy

- Pre-conception counselling should be addressed by all healthcare professionals, with referral to professionals with relevant experience as appropriate to optimize all therapy, including non-pharmacological options for chronic pain management during pregnancy (GRADE 1A, SOA 99.5).
- The risks and benefits of drug treatment to mother and foetus should be discussed and clearly documented by all healthcare professionals involved in the patient’s care (GRADE 1A, SOA 99).
- The cause of pain and other symptoms should be assessed and managed appropriately (GRADE 1B, SOA 98.5).
- The requirement for analgesia should be assessed and minimum effective dose should be prescribed and titrated according to response (GRADE 1B, SOA 100).
- Tricyclic antidepressants are preferred over other antidepressant medications to manage chronic pain (GRADE 1B, SOA 98.1).
- Cessation of anti-depressant therapy that is being used as chronic pain medication in the post-natal period is not recommended, due to the risk of adverse impact on mood (GRADE 1C, SOA 96).
- LDA (≤150 mg/day) is recommended in all patients at high risk for pre-eclampsia (GRADE 1A, SOA 99.5).
- Low molecular weight heparin (LMWH) is the preferred anticoagulant (GRADE 1A, SOA 100).
- Nifedipine is the preferred vasodilator (GRADE 1B, SOA 98.5).
- Paternal drug exposure may reduce male fertility but has not been associated with adverse foetal development or pregnancy outcome. Although evidence is weak, we recommend that men are reassured about the safety of fathering a pregnancy while taking medicines to manage comorbidities as described in this guideline (GRADE 1C, SOA 98).

### Pain management: conventional analgesics

#### Paracetamol

Two systematic reviews [16, 17] were selected. Overall, paracetamol was considered to have a favourable safety profile in pregnancy. Potential links between paracetamol use in pregnancy and pre-term birth and adverse neurodevelopmental outcomes (principally autism and attention deficit hyperactivity disorder) were confounded by maternal disease and selection bias [16]. UKTIS states that ‘In most studies, risk of abnormal neurodevelopment correlated with duration of paracetamol exposure. However, significant methodological limitations of these studies limit the conclusions that can be drawn, and a causal association remains unproven’ [18].

There were no convincing associations with congenital malformations. UKTIS notes that findings of a possible increased risk of cryptorchidism in male offspring following paracetamol use during pregnancy are conflicting [18]. Current Royal College of Obstetricians (RCOG) guidance is that paracetamol remains safe for use during pregnancy and breastfeeding, and its use in any trimester does not appear to increase the risk of major birth defects [19].

There were conflicting results on the risk of developing wheeze in infants exposed to paracetamol during pregnancy. A recent meta-analysis of 13 articles and 1 043 109 individuals identified a statistically significant association between prenatal paracetamol exposure and increased risk of child asthma [odds ratio (OR) 1.19; 95% CI, 1.12, 1.27; *P* < 0.00001] in a random-effect model [17]. This significant association was observed for first trimester as well as second and third trimester exposure.

There were no studies identified which specifically examined neonatal outcomes after drug exposure in breastmilk. LactMed [20] reports low amounts of this drug in breastmilk at levels much less than doses usually given to infants with few reports of adverse events. The following recommendations for paracetamol were developed based on evidence as shown in Supplementary Table S3, available at *Rheumatology* online.

##### Recommendations for paracetamol in pregnancy and breastfeeding

- Paracetamol is the analgesic of choice and compatible peri-conception and throughout pregnancy (GRADE 1B, SOA 99).
- LactMed describes paracetamol as a good choice for analgesia and fever reduction in breastfeeding mothers (GRADE 2C, SOA 99.5).

### Codeine

One systematic review [16] considered outcomes from opioid-exposed pregnancies, including three studies of congenital malformation in >20 000 codeine-exposed pregnancies with conflicting results. Of these three studies, a large Norwegian population-based cohort study [21] found no statistically significant associations between neonatal codeine exposure and congenital malformations. In contrast, a large case-control study found that first trimester exposure to opioids (codeine and hydrocodone in 69% of cases) was significantly associated with congenital heart defects, spina bifida and gastroschisis [22], although there was a high risk for recall bias in this study. A case-control study found a significant association between spina bifida in infants and first trimester maternal opioid use, although it was not with codeine use specifically and potential confounders such as illicit drug use/dependence and sociodemographic data exist in this study [23]. UKTIS notes that the methodological limitations of studies make it difficult to draw any firm conclusions and more robust research is needed before firm conclusions regarding the risk of congenital anomalies with codeine use can be provided [18].

No convincing associations between first trimester opioid use and miscarriage have been found. A large population study found increased rates of premature delivery, reduced birthweight and pre-eclampsia in codeine-exposed patients compared with controls that was considered to be due to confounding factors such as maternal disease and lack of adjustment for gestational age [21]. This study also found a significant increase in post-partum haemorrhage (18.3% *vs* 14.5%; OR 1.3; 95% CI 1.1, 1.5) in 2666 mothers exposed to codeine [21] and hypothesized it was due to an opioid effect weakening myometrial contraction, but the precise mechanism is unknown.

UKTIS describes theoretical concerns that maternal use of codeine near term may be associated with respiratory depression in the neonate and notes that the only study that has investigated the risk of neonatal respiratory depression found no increased risk [18]. The potential risk of neonatal abstinence syndrome (NAS) is lower with short courses of short-acting opioids and must be weighed against the benefits for treating acute pain that often outweigh risks. Prolonged opioid use, however, should be avoided and if used in the last trimester, neonatologists should be advised due to the risks of NAS [16].

Controversy remains over whether codeine is safe in breastfeeding. Central nervous system (CNS) depression was reported by mothers in 16.7% (35/210) babies exposed to codeine, compared with 0.5% exposed to paracetamol [24]. In the same study there was one neonatal death and high morphine levels were found at post-mortem. The mother had received high doses of codeine (>2 mg/kg/day) and was subsequently found to be an ultra-rapid CYP2D6 metaboliser. Another study demonstrated dose-dependent CNS depression in 24% (17/72) of infants exposed to codeine through breastmilk [25]. A large study of 7804 infants reported conflicting results, but specifically, there was no difference in poor Apgar scores, postnatal complications, admission to special care baby units, readmission to hospital, resuscitation or death in infants exposed and not exposed to codeine [26].

LactMed notes that numerous professional organizations and regulatory agencies recommend that other agents are preferred over codeine during breastfeeding but that other opioid alternatives have been less studied and may not be safer [20]. It is acknowledged that due to its unpredictable metabolism, administration of codeine results in delivery of an unknown quantity of morphine. Therefore, despite its widespread use and probable safety in most cases, we would advise caution with prolonged use of codeine in breastfeeding and appropriate advice to the mother to seek medical attention if she has any concerns regarding lethargy or drowsiness in her child. The following recommendations for codeine were based on evidence as shown in Supplementary Table S3, available at *Rheumatology* online.

#### Recommendations for codeine in pregnancy and breastfeeding

- Codeine is compatible peri-conception and throughout pregnancy, although long-term use should be avoided. There is no consistent evidence to recommend a dose reduction pre-delivery but neonatologists should be aware of maternal use (GRADE 1B, SOA 97.8).
- Caution is advised with use of codeine in breastfeeding, due to the risk of CNS depression resulting from unpredictable metabolism of codeine to morphine (GRADE 1C, SOA 98).

### Tramadol

A systematic review [16] considered outcomes from tramadol-exposed pregnancies. It included a large prospective cohort study of over 1.6 million women [27] that reported a statistically significant association between tramadol use in pregnancy and major congenital malformations (OR 1.33; 95% CI 1.05, 1.70) and cardiovascular defects (OR 1.56; 95% CI 1.04, 2.29), although it did not adjust for indication, duration or dose of medication.

UKTIS notes that a single study identified an increased risk of miscarriage among women who used tramadol in early pregnancy and recommended further studies considering the impact of confounding factors to clarify this finding. A single cohort study found no increased risk of preterm delivery within 146 pregnancies exposed to tramadol in at least the first trimester [18]. A small number of case reports have reported NAS with long-term intrauterine exposure to tramadol [28], although none have compared the relative rate of NAS with tramadol compared with other opioid analgesics.

LactMed states that the excretion of tramadol into milk is low and even lower amounts of the active metabolite, O-desmethyltramadol, are excreted and a study of breastfeeding in newborn infants found no adverse effects attributable to tramadol compared with controls [20]. There has been one death, however, in the 8-month-old breastfed infant of a woman addicted to tramadol, although the death was not definitely attributable to tramadol exposure in breastmilk [29]. Current RCOG advice states that tramadol can continue to be used (with caution) during breastfeeding and the lowest effective dose should be used for the shortest time possible [19]. The following recommendations for tramadol were based on evidence as shown in Supplementary Table S3, available at *Rheumatology* online.

#### Recommendations for tramadol in pregnancy and breastfeeding

- Avoid tramadol peri-conception and in first trimester and only consider in second/third trimester if no alternative analgesia (GRADE 2B, SOA 97.8).
- Based on limited data, tramadol may be compatible with short-term use in breastfeeding (GRADE 2C, SOA 94.8).

### Other treatments for chronic pain

#### Amitriptyline

NICE guidance [30] and a systematic review [16] described amitriptyline use in pregnancy. Notably, the evidence base underlying amitriptyline use in pregnancy comes from its use as a tricyclic antidepressant (TCA) to treat depression at doses of 150–300 mg and evidence of its use in pregnancy to treat chronic widespread pain at 75 mg per day or less is lacking. In addition, most studies report on TCAs as a drug class and although they include (some) women exposed to amitriptyline, they lack a separate assessment of their pregnancy outcomes so do not provide information about specific amitriptyline exposure [18].

Overall, no increased risk of congenital malformations has been found for TCAs [16]. UKTIS reports conflicting findings, with possible associations with spontaneous abortion, preterm delivery, and autism spectrum disorder identified in some (but not all) studies [18]. There is an association between discontinuation of antidepressants and a high risk for relapse of mood disorders that can adversely impact on pregnancy. It is unknown, however, whether a similar phenomenon of rebound pain exists when antidepressants used for analgesia are discontinued in pregnancy, particularly for amitriptyline that is used at 2–4-fold higher doses to treat depression compared with chronic pain. If a decision is made to stop amitriptyline in pregnancy, the dose should be tapered gradually where possible [16, 30].

Low levels of amitriptyline and its metabolites are reported in breastmilk with no adverse effects described with limited follow-up, summarised in LactMed [20]. The following recommendations were based on evidence as shown in Supplementary Table S4, available at *Rheumatology* online.

##### Recommendations for amitriptyline in pregnancy and breastfeeding

- Amitriptyline is compatible with pregnancy. There is no evidence of adverse effect on IQ or developmental outcomes (GRADE 1C, SOA 100).
- Because very little amitriptyline is found in breastmilk with antidepressant doses and it is used at lower doses for chronic pain, it is unlikely to cause adverse effects in breastfed infants (GRADE 1C, SOA 100).

### Gabapentin and pregabalin

Data on gabapentin use in pregnancy comes mostly from studies of the treatment of maternal epilepsy at doses generally higher that those used to treat chronic pain, while information on pregabalin use in pregnancy comes from studies of its use to treat neuropathic pain [18]. A systematic review [16] of gabapentin use in pregnancy to treat epilepsy did not find any evidence of an increased rate of major malformations or other adverse outcomes attributable to gabapentin exposure. No long-term outcomes were reported. Furthermore, a systematic review and meta-analysis published in abstract form only [31], of eight cohort studies (four prospective and four retrospective), reporting 5 072 286 unexposed and 949 exposed pregnancies found that first trimester exposure to gabapentin was not statistically significantly associated with major congenital malformations (OR 0.83; 95% CI 0.45, 1.53).

A prospective cohort study using Teratology Information Services data from seven European countries [32] found that first trimester use of pregabalin was associated with significantly higher rates of major congenital malformations when compared with unexposed pregnancies (OR 3.0; 95% CI 1.2, 7.9). This study, however, was limited by a small sample size (164 exposed pregnancies) and lack of adjustment for potential confounding by use of concomitant medications [16]. A systematic review of pregabalin use, mostly to treat neuropathic pain in pregnancy, including data from this study and two others identified 651 pregnancy exposures and concluded that pregabalin exposure during pregnancy is not devoid of structural teratogenicity potential [33]. UKTIS reviewed data from six controlled studies, with outcomes of >3000 exposed pregnancies of patients with chronic pain or other non-epilepsy indications. They conclude that this data does not indicate that maternal pregabalin use in pregnancy is associated with increased risks of malformation, miscarriage or adversely affects foetal growth [18]. Interestingly, a systematic review and meta-analysis published in abstract form only [34], of six studies reporting 2319 exposed pregnancies and 4 982 778 unexposed pregnancies found that first trimester exposure to pregabalin was not significantly associated with an increased risk of major congenital malformations (OR 1.20; 95% CI 0.92, 1.57).

Following completion of our guidelines, the MHRA issued a safety warning on the use of pregabalin in pregnancy based on new data available online [35]. This new data has been considered in the latest UKTIS update in April 2022 that states that the available data do not provide conclusive evidence that maternal pregabalin use in the first trimester, or at any stage of pregnancy, is associated with increased risks of either overall malformation or any specific malformations. Therefore, our recommendations have not changed.

UKTIS found no controlled studies of the risk of neonatal complication following prenatal gabapentin or pregabalin exposure, although one study has described a small number of affected infants, including one case of neonatal withdrawal following *in-utero* pregabalin exposure [18]. Use of any centrally acting drug throughout pregnancy or near delivery may be associated with withdrawal symptoms in the neonate and/or NAS.

Although evidence is lacking and it is uncertain if pregabalin or gabapentin impact upon maternal folate status, UK guidelines state that women who take any anti-epileptic medication should be prescribed high-dose folic acid (5 mg/day) preconceptually and in the first trimester [19].

There remains limited data on use of these drugs in breastfeeding. According to LactMed, low levels of gabapentin and pregabalin have been found in breastmilk with no adverse effects on infants reported in limited case reports/series (*n* < 10) for gabapentin. Therefore, both drugs may be considered if required by the breastfeeding mother [20]. The following recommendations were based on evidence as shown in Supplementary Table S4, available at *Rheumatology* online.

#### Recommendations for gabapentin and pregabalin in pregnancy and breastfeeding

- Gabapentin at lowest effective dose may be considered in pregnancy with folic acid supplementation if no alternative analgesic is suitable (GRADE 1B, SOA 95).
- Gabapentin may be considered in breastfeeding if no alternative analgesic is suitable (GRADE 2C, SOA 96).
- Pregabalin may be considered in pregnancy (with folic acid supplementation) and during breastfeeding (GRADE 2C, SOA 95.3).

### Serotonin–norepinephrine reuptake inhibitors (SNRIs)

NICE guidance [30] and two systematic reviews [16, 33] described SNRI use in pregnancy. There was no association between first trimester exposure to venlafaxine and an increased risk for major congenital malformations [16]. UKTIS notes that although there are some reports of an increased risk of miscarriage following gestational exposure to venlafaxine, the data are inconsistent and likely confounded by indication and other factors [18]. Some studies have found a possible association with an increased risk for some perinatal complications, including a withdrawal syndrome with venlafaxine use in the third trimester [16]. A more limited data set for duloxetine does not suggest a clinically important increased risk for major malformations but has identified prenatal antidepressant exposure syndrome in two of five case reports and conflicting reports of increased rates of gestational hypertension and spontaneous abortion [33].

LactMed reports that infants receive venlafaxine and its active metabolite in breastmilk, and the metabolite of the drug can be found in the plasma of most breastfed infants, but no proven drug-related side effects have been reported in small case series. Little published information is available on the use of duloxetine during breastfeeding; however, the dose in milk is low and serum levels were low in two breastfed infants [20]. The following recommendations were based on evidence as shown in Supplementary Table S4, available at *Rheumatology* online.

#### Recommendations for SNRIs in pregnancy and breastfeeding

- Venlafaxine is compatible at conception and throughout pregnancy. There may be an increased risk of neonatal abstinence syndrome/short-term behavioural effects, but larger studies are needed to evaluate this finding (GRADE 2C, SOA 95.8).
- Duloxetine may be considered in pregnancy and breastfeeding but there are fewer data than for venlafaxine (GRADE 2C, SOA 95.3).
- Venlafaxine and duloxetine may be considered in breastfeeding if there is no alternative chronic pain medication (GRADE 2C, SOA 95.8).

### Selective serotonin reuptake inhibitors (SSRIs)

NICE guidance [30] and a systematic review [16] described SSRI use to treat depression in pregnancy. The SSRIs are used to treat chronic pain at similar doses used to treat depression. A systematic review considered data from >50 000 infants exposed to SSRIs *in utero*, which did not show an overall increased risk for congenital malformations [16]. It describes ongoing debate about the risk for cardiovascular malformations with first-trimester use of SSRIs with risk found from some [36, 37] but not all studies [38, 39]. Overall, any increase in absolute risk was thought unlikely to be clinically significant and may be associated with particular SSRIs, principally fluoxetine and paroxetine [36, 37].

The UKTIS summary of findings to date from studies on SSRIs states that a causal association between use of SSRIs in pregnancy and any type of congenital malformation has not been confirmed. It also describes conflicting results from other outcomes and concludes that available data do not suggest that SSRI use in pregnancy increases the risk of stillbirth and that possible associations with neurodevelopmental impairment in infants requires further study. An increased risk, however, of persistent pulmonary hypertension (PPHN) of the newborn has also been reported following exposure to SSRIs as a class beyond 20 weeks of gestation and, although it remains an uncommon event (0.2–1.2% *vs* 0.1–0.2% in the background population), it represents a potentially serious neonatal complication [18]. Because there is no robust evidence of a superior safety profile for any one drug, switching between drugs is not recommended if depression is stable on treatment.

NICE recommendations on use of TCAs, SSRIs or SNRIs include consideration of: the uncertainty about whether any increased risk to the foetus and other problems for the woman or baby can be attributed directly to these drugs or may be caused by other factors; and the risk of discontinuation symptoms in the woman and neonatal adaptation syndrome in the baby with most TCAs, SSRIs and (S)NRIs, in particular paroxetine and venlafaxine [30].

There is limited information on the use of these drugs in breastfeeding. One small study showed temporarily reduced growth during exposure to fluoxetine in breastmilk. There have been no studies specifically investigating compatibility of paroxetine and sertraline with breastfeeding, but sertraline is reported as having one of the lowest rates of transmission to breastmilk [20]. The following recommendations were based on evidence as shown in Supplementary Table S4, available at *Rheumatology* online.

#### Recommendations for SSRIs in pregnancy and breastfeeding

- Fluoxetine, paroxetine and sertraline are compatible with pregnancy (GRADE 1B, SOA 98.8).
- Based on limited evidence, SSRIs are compatible with breastfeeding (GRADE 2C, SOA 98.3).

### NSAIDs and anti-platelet drugs

Non-selective cyclooxygenase (COX) inhibitors have different indications in pregnancy. NSAIDs have analgesic and anti-inflammatory actions mediated through peripheral inhibition and differential selectivity of COX enzymes. In contrast, LDA at doses ≤150 mg/day is used to prevent thrombosis and pre-eclampsia in high-risk groups throughout pregnancy in patients with rheumatic diseases. Clopidogrel is an anti-platelet agent that may sometimes be used with or instead of LDA to reduce cardiovascular risk. NICE guidance [40], four systematic reviews [16, 41–43] and a case report/review [44] evaluated LDA, NSAID and clopidogrel use in pregnancy.

### NSAIDs and COX-2 inhibitors

Overall, there is no consistent evidence for an increased risk of teratogenic effects with NSAID use in pregnancy. There are mixed findings regarding a potential increased risk for miscarriage, with findings limited by methodology and larger associations reported for indomethacin and diclofenac use in the periconceptional period [16]. There was no information on safety of COX2 inhibitors in pregnancy. NSAIDs are reported to increase the incidence of luteinized unruptured follicle (LUF) syndrome, whereby an anovulatory cycle results due to failure of normal follicular wall rupture despite normal ovarian follicular development and elevation of serum progesterone. COX-2 is active in the ovaries during follicular development; thus, inhibition via COX-2 inhibitors is thought to result in LUF. Although similar findings have been reported for both COX-1 and COX-2 NSAIDs, the risks have been found to be greater in patients with inactive disease and in those taking a COX-2 inhibitor (etoricoxib) rather than non-selective NSAIDs and it is reversible, following drug withdrawal [16].

A systematic review of the effects of various drugs on foetal cardiac function evaluated by ultrasound found that all NSAIDs (including COX2 inhibitors) increased constriction in the ductus arteriosus, within 4–30 h of exposure and resolved by 72 h of discontinuation [43]. In this study the critical gestational age (measured for indomethacin) increased from 5–10% of foetuses at weeks 26–27 to 50% at week 32.

In 2020 the United States Food and Drug Administration (FDA) recommended that all NSAIDs be avoided from gestational week 20 rather than the previously advised 30 weeks. This advice was based on their updated review of published data to 2016 and 35 cases reported to FDA identifying an increased risk of oligohydramnios and renal impairment that began at 20 weeks of gestation and were mostly reversible on stopping NSAID. They clarify their advice for healthcare professionals, stating that the use of NSAIDs between 20 and 30 weeks of pregnancy should be limited to the lowest effective dose for the shortest duration [45].

### Low-dose aspirin

LDA has been extensively studied and shown to improve outcomes in high-risk pregnancies. A Cochrane review of 77 trials, involving 40 249 women and their babies, found high quality evidence that antiplatelet agents (mostly LDA up to 150 mg/day) reduced pre-eclampsia and its complications [42]. A systematic review of 22 RCTS of LDA plus heparin compared with other treatments in patients with APS, including 1515 treatment and 1531 control subjects, found that adverse pregnancy outcomes were significantly improved with LDA and heparin [41]. The use of LDA in the third trimester of pregnancy is not associated with premature closure of ductus arteriosus and NICE guidelines for management of hypertension in pregnancy advises treatment with LDA until delivery [40].

### Clopidogrel

There was no demonstrable maternofoetal toxicity in 13 (mostly second and third trimester only) pregnancy exposures to clopidogrel [44]. UKTIS does not report on clopidogrel.

LactMed considers various non-selective NSAIDs to be acceptable during breastfeeding and prefers ibuprofen because of its extremely low levels in breastmilk, short half-life and safe use in infants in doses much higher than those excreted in breastmilk, as an analgesic or anti-inflammatory agent in breastfeeding mothers. There was no information on COX2 inhibitors. Aspirin doses up to 325 mg daily are not excreted into breastmilk so LDA may be considered as an antiplatelet drug for use in breastfeeding women and is preferred to clopidogrel as no information is available on this drug [20]. Recommendations for these drugs were based on evidence as shown in Supplementary Tables S5 and S6, available at *Rheumatology* online.

#### Recommendations for NSAIDs and COX-2 inhibitors in pregnancy and breastfeeding

- Discordant findings from retrospective, large studies with population controls on the use of non-selective NSAIDs in the first trimester of pregnancy raise the possibility of a low risk of miscarriage and malformation. Therefore, these drugs should only be used intermittently in the first trimester of pregnancy (GRADE 1B, SOA 97.3).
- Intermittent rather than regular use of all non-selective NSAIDs except LDA is recommended throughout pregnancy and weaned from end of second trimester (26 weeks) to stop by gestational week 30 to avoid premature closure of the ductus arteriosus (GRADE 1B, SOA 98).
- At present there are limited data on selective cyclooxygenase-2 inhibitors; they should therefore be avoided during pregnancy (GRADE 2C, SOA 98.5).
- Non-selective NSAIDs (especially ibuprofen) are compatible with breastfeeding (GRADE 1C, SOA 98.8).

#### Recommendations for LDA and clopidogrel in pregnancy and breastfeeding

- LDA may be continued throughout pregnancy and NICE guidelines (2019) for hypertension in pregnancy advise treatment with LDA (for prophylaxis of pre-eclampsia) until delivery (GRADE 1B, SOA 99.0).
- LDA is compatible with breastfeeding (GRADE 2C, SOA 99.8).
- There are limited data on clopidogrel but it may be considered where alternative drugs are not suitable in pregnancy and breastfeeding (GRADE 2C, SOA 96.3).

### Colchicine and dapsone

These drugs were not considered in the previous BSR guideline and are now included because they may be used to treat certain inflammatory rheumatic diseases. UKTIS does not report on colchicine or dapsone. A systematic review and meta-analysis of colchicine use in 550 pregnancies of women with mostly familial Mediterranean fever (FMF) at doses of 1–2 mg per day, compared with 1263 non-exposed pregnancies found this drug did not significantly increase the incidence of foetal malformations or miscarriage when taken during pregnancy [46]. However, the National Amyloidosis Centre recommends to continue the prescribed dose as there are no established safety concerns at colchicine doses >2 mg daily during pregnancy [47].

No systematic review data was identified for dapsone. A review of 924 pregnancies exposed to dapsone to treat malaria was precluded from meaningful risk-benefit analysis due to limited reporting of outcomes [48]. They concluded that the use of dapsone may be considered when no suitable alternative is available and the threat of malaria is the greater risk. It is a safe option in pregnant patients without glucose-6-phosphate dehydrogenase (G6PDH) deficiency and can be used during lactation while monitoring the baby for haemolysis and G6PDH deficiency [49]. A review of the treatment of rheumatic and autoimmune skin disease in women during pregnancy concluded that dapsone may be safely and cautiously used during pregnancy [50].

LactMed reports that long-term prophylactic maternal doses of colchicine up to 1.5 mg daily produce levels in milk that result in the infant receiving <10% of the maternal weight-adjusted dosage and no adverse effects have been reported from limited studies. It also states that the highest milk levels occur 2–4 h after a dose, so avoiding breastfeeding during this time can minimize the infant dose, or simply taking the drug after nursing. LactMed also states that dapsone can be used during breastfeeding; however, haemolytic anaemia might occur, especially in newborn infants and in those with G6PDH deficiency [20]. Recommendations for these drugs were based on evidence as shown in Supplementary Table S5, available at *Rheumatology* online.

#### Recommendations for colchicine and dapsone in pregnancy and breastfeeding

- Colchicine therapy may be considered during pregnancy (GRADE 1B, SOA 99.5).
- Dapsone may be used in pregnancy (GRADE 2C, SOA 95.0).
- Colchicine may be used in breastfeeding (GRADE 2C, SOA 98.3).
- Dapsone may be used in breastfeeding and due to the risk of haemolytic anaemia it is advised to monitor the infant for signs of haemolysis, especially in newborn or premature breastfed infants (GRADE 2C, SOA 90.7).

### Anticoagulants in rheumatic disease

The deleterious effects of warfarin and compatibility of heparin in pregnancy are well described and evidence-based guidelines for the management of venous thromboembolism (VTE) and thrombophilia in pregnancy exist [51].

### Heparin

A systematic review [41] and systematically produced guidelines [52] describe the utility of heparin in the management of VTE and pregnancy morbidity in pregnant patients with antiphospholipid syndrome (APS). Heparin/LMWH does not cross the placenta [1, 2].

Heparins are compatible with breastfeeding. There were no additional studies identified, but LactMed states that no particular caution is required as the molecular weight of heparin is such that it is unlikely to be appreciably excreted into breastmilk.

### Warfarin

Warfarin has the ability to cross the placenta and is associated with an increased risk of congenital abnormalities including a characteristic warfarin embryopathy (hypoplasia of the nasal bridge, congenital heart defects, ventriculomegaly, agenesis of the corpus callosum, stippled epiphyses) in ∼5% of foetuses exposed between 6 and 12 weeks of gestation. Warfarin should therefore be avoided between 6 and 12 weeks [51]. While heparin/LMWH remains the anticoagulant of choice in pregnancy for the majority of patients considered to be at increased thrombotic risk in pregnancy, warfarin may be considered in pregnancy for women with mechanical heart valves (MHVs). The LMWH regimen could be used for those who refuse warfarin and other vitamin K antagonists (VKAs) [53]. In management of atrial fibrillation, VKAs may be used after the first trimester [54].

LactMed is reassuring both with regard to low drug levels in breastmilk and infant serum, and no reported adverse effects.

### New anticoagulants

A systematic review [55] identified 236 cases of direct oral anticoagulants (DOAC) use in pregnancy of rivaroxaban (*n* = 178), dabigatran (*n* = 27), apixaban (*n* = 21) and edoxaban (*n* = 10). DOACs were mostly used for prophylaxis or treatment of venous thromboembolism (*n* = 91). DOACs were discontinued within the first 2 months of pregnancy in 84%, and the maximum reported duration of use was 26 weeks. Pregnancy outcome data were available for 140 pregnancies. Thirty-nine pregnancies were electively terminated. In the remaining 101 pregnancies, total miscarriage rate was 31% (*n* = 31) and live birth rate was 68% (*n* = 69, one missing). Foetal and neonatal abnormalities were reported in eight pregnancies, of which at least half were suspected to be related to rivaroxaban use during the first trimester of pregnancy. In only 18% of cases (*n* = 42), the presence or absence of thrombotic and bleeding complications was reported. This limited evidence raises concern regarding embryo-foetal safety, with a high incidence of miscarriages and a 4% rate of congenital anomalies with the use of rivaroxaban. Overall, not enough data are available to judge safety and efficacy of the use of DOACs during pregnancy and rivaroxaban and dabigatran have both been shown to cause adverse obstetric outcomes in animal studies, described in summary of product characteristics [56, 57]. UKTIS does not report on DOACs.

LactMed describes several case reports that consistently indicate that maternal doses of rivaroxaban of 15–30 mg daily produce low levels in milk that are considerably below doses required for anticoagulation in infants [20]. Therefore, breastfeeding is not contraindicated if rivaroxaban is required by the mother. There are no data on the excretion of dabigatran into human milk.

### Fondaparinux

Fondaparinux may be considered if there is an allergy or adverse response to LMWH, although solid data are lacking and minor transplacental passage has been demonstrated, without any adverse materno-foetal effects in five pregnancies [58]. LactMed considers use of fondaparinux to be acceptable during breastfeeding [20]. Recommendations on all anticoagulants were based on evidence as shown in Supplementary Table S6, available at *Rheumatology* online.

#### Recommendations for anticoagulants in pregnancy and breastfeeding

- LMWH heparin is compatible throughout pregnancy (GRADE 1A, SOA 100).
- LMWH is compatible with breastfeeding (GRADE 1C, SOA 100).
- The use of warfarin in pregnancy is associated with increased foetal risk throughout pregnancy and has limited indications, therefore should only be considered in exceptional circumstances (GRADE 1B, SOA 98.8).
- Warfarin is compatible with breastfeeding (GRADE 1A, SOA 100).
- Direct oral anticoagulants (DOACs) cannot be recommended in pregnancy (GRADE 1C, SOA 97.9).
- Rivaroxaban may be considered in breastfeeding (GRADE 2C, SOA 95.3).
- Other DOACs are not recommended in breastfeeding due to lack of human data and concerns from animal studies (GRADE 1C, SOA 97.4).
- Fondaparinux may be considered in pregnancy and breastfeeding if there is an allergy or adverse response to LMWH (GRADE 2C, SOA 95.5).

### Bisphosphonates

Bisphosphonates are not ideal in women planning pregnancy because the absolute risk of fracture is small in this age group and the skeletal half-life of these drugs is very long. The number of human pregnancy exposures remains limited and a detailed literature review identified 40 pregnancies [59], while a systematic review published in abstract form described outcomes from 120 bisphosphonate-exposed pregnancies [60]. Overall, rates of congenital malformation and miscarriages were comparable in bisphosphonate and controls, although limiting factors included: few comparator groups; heterogeneous maternal disease; concomitant medication; and small sample size. Controlled studies have demonstrated possible associations between preconceptual/gestational bisphosphonate exposure and increased risk of spontaneous abortion, decreased infant birth weight, and lower gestational age at delivery. These findings, however, may reflect data limitations and/or uncontrolled confounding (UKTIS). Therefore, further controlled studies are required to fully establish the safety of bisphosphonates in pregnancy and they are not drugs of choice in women planning pregnancy.

LactMed states that limited evidence indicates that breastfeeding after cessation of long-term bisphosphonate treatment appears to have no adverse effects on the infant. There is no information on the use of alendronate or risedronate during breastfeeding. Limited information indicates that maternal doses of pamidronate of 30 mg intravenously produce very low levels in milk and because pamidronate has a serum half-life of ∼3 h, is highly bound to calcium and poorly absorbed orally, absorption of pamidronate by a breastfed infant is unlikely. Therefore, withholding breastfeeding for 12–24 h after a dose should ensure that the breastfed infant is exposed to little or no pamidronate. If the mother receives a bisphosphonate during pregnancy or nursing, some experts recommend monitoring the infant’s serum calcium during the first 2 months postpartum [20]. These recommendations were based on evidence as shown in Supplementary Table S7, available at *Rheumatology* online.

#### Recommendations for bisphosphonates in pregnancy and breastfeeding

- There is insufficient data upon which to recommend bisphosphonates in pregnancy or to advise a specific time for them to be stopped pre-conception. Given their biological half-life in bone of up to 10 years and no evidence of harm from limited reports of their use in pregnancy, a pragmatic recommendation is that they should be stopped 3 months in advance of pregnancy (GRADE 2C, SOA 96.8).
- There are no data on which to base a recommendation for the use of bisphosphonates during breastfeeding (GRADE 2C, SOA 98.5).

### Antihypertensive medication in rheumatic disease

Patients with ARD, particularly renal SLE and systemic sclerosis (SSc) frequently require anti-hypertensive treatment for sometimes life-threatening disease, such as scleroderma renal crisis, that may require specialist use of certain anti-hypertensive drugs. The introduction of ACEis for the treatment of SSc renal crisis has significantly reduced mortality rates of up to 50% in the 1970s to a mortality of up to 20% at 6 months after introducing ACEis. The use of ACEis may therefore be indicated (also in pregnancy) in exceptional circumstances [7].

The management of pre-existing and new onset hypertension in pregnancy has been comprehensively reviewed and updated in the 2019 NICE guideline, Hypertension in Pregnancy: Diagnosis and Management [40].

### Angiotensin blockade

Disruption of the renin-angiotensin system (RAS) in pregnancy by maternal treatment with ACEis or angiotensin receptor blockers (ARBs) in the second/third trimester leads to abnormal foetal renal development, known as foetal RAS blockade syndrome [61]. There are conflicting results, however, on the risk of this fetopathy occurring after first trimester exposure. A systematic review and meta-analysis [62] of 19 articles involving 4 163 753 pregnant women found a significant association between overall congenital malformations and first trimester‐only exposure to ACEis/ARBs (odds ratio 1.94; 95% CI 1.71, 2.21; *P* <0.0001). This review also found a significant association between cardiovascular malformations, miscarriage and stillbirth and ACEi/ARB exposure. A similar risk was observed in a cohort of 1 333 624 pregnancies [63], including 4107 (0.31%) following first trimester ACEi exposure that found an increased risk of overall malformations in the ACEi-exposed pregnancies [unadjusted relative risk (RR), 1.82; 95% CI 1.61, 2.06] and of cardiac malformations (RR 2.95; 95% CI 2.50, 3.47). On further analysis, however, restricting the cohort to pregnancies complicated by chronic hypertension (both exposed and unexposed) and accounting for other confounding factors, there was no significant increase in the risk of any of the outcomes assessed.

NICE guidelines state that women taking ACEis/ARBs should be advised of the increased risk of congenital anomalies if these drugs are taken during pregnancy and to discuss alternative antihypertensive drugs with their clinician responsible for managing their condition [40]. If they become pregnant on ACEis/ARBs they should be stopped (preferably within 2 working days of notification) and other antihypertensive treatments offered. UKTIS recommends that where prolonged first trimester exposure has occurred, a 20-week anomaly scan should focus on cardiovascular, renal and neurological development, in addition to the routine anatomical checks. UKTIS states that ACEi fetopathy following exposure to ACEis in the second and third trimesters of pregnancy is well-described and may include oligohydramnios, renal tubular dysgenesis, neonatal anuria, hypocalvaria, pulmonary hypoplasia, persistent patent ductus arteriosus, mild-to-severe intrauterine growth restriction, and foetal or neonatal death. It is proposed that these effects occur as a result of a direct effect on the foetal RAS which begins to function from ∼26 weeks gestation. A small prospective case series has suggested that the risk period for ACEi fetopathy is with exposure beyond 20 weeks gestation. Due to data limitations, the absolute risk of ACEi fetopathy is unclear. Due to the risk of ACEi fetopathy, use of ACEis in the second and third trimesters is generally contraindicated and should only be reserved for cases of severe maternal illness that cannot be managed using alternative drugs [18].

Negligible amounts of enalapril and captopril are transferred into breastmilk with no adverse effects reported on the breastfed infants of mothers treated with short-acting ACEis [64]. NICE states that enalapril may be offered to treat hypertension in breastfeeding mothers with appropriate monitoring of maternal renal function and serum potassium [40]. The following recommendations were based on evidence as shown in Supplementary Table S8, available at *Rheumatology* online.

#### Recommendations for ACEis/ARBs in pregnancy and breastfeeding

- ACEis and ARBs should be stopped as soon as possible when pregnancy is confirmed in the first trimester and if necessary an alternative antihypertensive compatible with pregnancy given (GRADE 1A, SOA 100).
- ACEis/ARBs should be avoided in the second and third trimester but may be considered under specialist advice in certain circumstances (GRADE 1C, SOA 98.5).
- Based on limited evidence, enalapril is compatible with breastfeeding (GRADE 2C, SOA 98.5).

### Calcium channel blockers

Calcium channel blockers (CCBs) including amlodipine, diltiazem, felodipine, lacidipine, lercanidipine, nicardipine, nifedipine, nimodipine and verapamil are mainly used for the treatment and prophylaxis of angina, and the treatment of hypertension where an ACEi/ARB is unsuitable. In patients with rheumatic disease, nifedipine or amlodipine are also used to treat Raynaud’s phenomenon.

UKTIS has not identified an increased risk of congenital malformations or other adverse pregnancy outcomes with CCBs, although data remains too limited to draw firm conclusions on many outcomes. Data on rates of preterm delivery, foetal growth and neurodevelopmental outcomes are too limited and/or confounded to permit an accurate risk assessment, but where a CCB is required to treat maternal hypertension or as a tocolytic, foetal benefits of use are likely to outweigh any unspecified risk and treatment should not be withheld on this basis [18]. Data is more limited for amlodipine so it is not included in alternatives to treat hypertension in pregnancy that include labetalol, nifedipine or methyldopa in order of preference [40].

LactMed describes low levels of nifedipine and amlodipine in breastmilk, without any adverse effects being reported among exposed infants [20]. NICE guidance of other antihypertensive drugs that may be offered in breastfeeding mothers include nifedipine, amlodipine, atenolol or labetolol [40]. Recommendations were based on evidence as shown in Supplementary Table S8, available at *Rheumatology* online.

#### Recommendations for CCBs in pregnancy and breastfeeding

- Nifedipine is compatible with pregnancy with no direct evidence of harm at doses up to 90 mg/day (GRADE 1A, SOA 99.0).
- Nifedipine is compatible with breastfeeding (GRADE 1B, SOA 100).
- Amlodipine can be considered in pregnancy and breastfeeding as there is no evidence of harm (GRADE 1C, SOA 97.9).

### Pulmonary vasodilators

Moderate-to-severe pulmonary hypertension (PHT) is a rare complication of certain ARDs and remains a contraindication to planned pregnancy with high mortality. Unintentional pregnancy and/or patient choice, however, means that treatment of this condition with specific pulmonary vasodilators may be required in pregnancy. Limited information on use of these drugs in human pregnancy was identified in the previous BSR guideline [2]. No studies were identified examining pregnancy outcomes after paternal exposure to any of these pulmonary vasodilators.

### Sildenafil

Sildenafil has been studied in the context of trying to improve utero-placental circulation in pregnancies affected by severe foetal growth restriction. A systematic review and meta-analysis were identified, examining the utility of sildenafil being used for treatment or prevention of obstetric diseases compared with placebo. They analysed 598 pregnant women from seven clinical trials with pre-eclampsia (*n* = 139), intrauterine growth restriction (*n* = 275) and oligohydramnios (*n* = 184) and found a significant improvement in birthweight following sildenafil treatment during pregnancy, with no difference in other outcomes [65].

However, in 2018 a randomised controlled study looking at using sildenafil to treat pregnant women in whom there was significant foetal growth restriction was halted early due to a number of neonates having persistent pulmonary hypertension of the newborn (PPHN). This multi-centered study enrolled women with a singleton pregnancy between 18 and 30 weeks with severe foetal growth restriction of likely placental origin, where the likelihood of perinatal death/severe morbidity was estimated to be significant. Participants were randomised into sildenafil or placebo arms. One of three study sites reported that PPHN appeared to be more prevalent in infants exposed *in utero* to sildenafil compared with placebo-exposed infants (*n* = 17/93 *vs n* = 3/90), that death among infants with PPHN was more common following sildenafil exposure (*n* = 11/17 *vs n* = 0/3), and that when the overall neonatal death rate was considered, there was a non-statistically significant trend towards an increased risk following sildenafil exposure (*n* = 19/93 *vs n* = 9/90). As a result, the trial was halted early and sildenafil is no longer recommended to improve placental function in severely growth-restricted babies [66, 67].

However, it should be noted that two further study sites for this trial did not detect an increased risk of PPHN and overall, this trial has not identified a clear beneficial effect of sildenafil on foetal outcome. PPHN is more prevalent in premature and growth-restricted foetuses and is a relatively rare complication in healthy babies delivered at term.

UKTIS recommends that general use of sildenafil in pregnancy should be avoided where possible [18]. However, in the context of maternal pulmonary hypertension there is clear benefit in the use of sildenafil to reduce the effects of pulmonary hypertension, which often gets worse during pregnancy. Case studies/case series data suggest that sildenafil exposure was not associated with miscarriage or congenital anomaly; however, the data is extremely limited (around 18 reported pregnancies). The risks and benefits of continuing sildenafil should be discussed with the patients, but most will likely require ongoing treatment.

LactMed describes limited data showing that sildenafil and its active metabolite are poorly excreted into breastmilk and amounts ingested by the infant are small and would not be expected to cause any adverse effects in breastfed infants [20].

### Bosentan

Animal data have revealed teratogenicity due to bosentan, including malformations of the head, mouth, face and large blood vessels in addition to an increased number of stillbirths and increased mortality [68]. Previously, we identified data from 12 pregnancies of women with PHT treated with bosentan in pregnancy plus multiple other medications, including sildenafil and iloprost, with reduced pregnancy duration of 37 weeks in one and reduced birth weight in two cases but no other maternal complications or foetal loss [2]. We identified one further case report of a patient with Eisenmenger syndrome exposed to long-term bosentan before and during pregnancy that was delivered by caesarean section at 27 weeks due to severe maternal PHT without any evidence of teratogenic effects of bosentan [69]. UKTIS does not report on bosentan.

LactMed states there is little published experience with bosentan during breastfeeding and an alternate drug may be preferred, especially while nursing a newborn or preterm infant [20].

### Prostacyclines

Previously we identified data on 23 pregnancies of patients with PHT (three with SLE) treated with iloprost (*n* = 5 pregnancies) or epoprostenol (*n* = 15 pregnancies) and three other prostacyclines (unspecified type) in patients who were taking multiple other medications, including immunosuppressants, sildenafil and bosentan [2]. Findings of premature deliveries and reduced birthweight were confounded by maternal disease. Furthermore, maternal complications were attributable to PHT. We did not identify any new evidence and UKTIS and LactMed do not report on iloprost or epoprostenol. The recommendations were based on evidence as shown in Supplementary Table S7, available at *Rheumatology* online.

#### Recommendations for pulmonary vasodilators in pregnancy and breastfeeding

- Established moderate to severe PHT remains a contraindication to pregnancy. If pregnancy occurs, the use of these pulmonary vasodilator drugs in pregnancy should be considered only as part of a multidisciplinary team assessment (GRADE 1C, SOA 99.5).
- Limited evidence supports the use of prostacyclines to treat PHT during pregnancy (GRADE 2C, SOA 98.0).
- Limited evidence supports the use of sildenafil to treat PHT during pregnancy (GRADE 2C, SOA 98.0).
- Bosentan is teratogenic in animals and although there is no evidence of harm from human pregnancy, the evidence is insufficient to recommend in pregnancy (GRADE 1C, SOA 98.8).
- There are no data relating to breastfeeding exposure to pulmonary vasodilators on which to base a recommendation (GRADE 2C, SOA 98.8).

### Paternal exposures

There remains limited data on paternal exposure to drugs used to treat rheumatic disease and reports of teratogenic effects linked with paternal exposure are lacking for all drugs considered in this guideline. Our updated search revealed the following additional information.

Links with adverse neurodevelopmental outcomes [70] but not asthma [71] have been reported following paternal exposure to paracetamol from the largest cohort study of paracetamol in pregnancy. A direct causal effect, however, remains unproven.

An observational prospective cohort study from Sweden [72] of 3983 children born to fathers receiving antidepressant treatment around conception, included fathers treated with: SSRIs, *n* = 2865; SNRIs, *n* = 470; and TCAs, *n* = 240. This study found paternal intake of all antidepressants studied to be safe with respect to the risk of preterm birth, malformation, autism or intellectual disability.

A systematic review of the effect of paternal exposure to immunosuppressive drugs on sexual function, reproductive hormones, fertility, pregnancy and offspring outcomes [73] found very weak evidence of reduced sperm parameters with codeine, tramadol and CCBs but not NSAIDs, LDA or ACEi and no abnormalities in offspring were reported for any drug. Interestingly, low-dose lisinopril (2.5 mg/day) has been shown to increase total sperm count and motility in a randomised, controlled, crossover pilot of study of normotensive men with idiopathic oligospermia leading to an unassisted pregnancy rate of 48.5% [74]. Small studies of chronic use of NSAIDs in men with rheumatic disease have not indicated any impairment of spermatogenesis and no evidence for harmful effects of NSAIDs on offspring [75].

A systematic review [76] of eight studies including 166 cases of paternal exposure found inconsistent reports of adverse effects of colchicine on sperm quality and only one study (*n* = 53) of paternal exposure did not find an adverse effect on offspring [77].

The following recommendations were based on evidence as shown in Supplementary Table S9, available at *Rheumatology* online.

#### Recommendations for paternal exposure

- Paracetamol is compatible with paternal exposure (GRADE 1B, SOA 98.5).
- Amitriptyline, SNRIs and SSRIs are compatible with paternal exposure (GRADE 1B, SOA 98.5).
- Non-selective NSAIDs are compatible with paternal exposure (GRADE 1C, SOA 98.4).
- Based on limited or no data and no association with adverse foetal development or pregnancy outcome, paternal exposure to all other drugs described in this guideline are unlikely to be harmful (GRADE 2C, SOA 97.3).

## Applicability and utility

### Implementation

Awareness of these guidelines will aid clinical practitioners and patients in decision making and will be raised through presentation at local, regional and national meetings. No barriers to implementation of these guidelines are anticipated.

### Key standards of care

Patients with rheumatic disease should receive tailored pre-pregnancy counselling and then be reviewed during pregnancy and the four month post-partum period by clinical practitioners with expertise in the management of rheumatic disease in pregnancy, in addition to their routine obstetric care. They should have access to written information on relevant medications in pregnancy and breastfeeding that is accurate and allows them to make informed decisions regarding compatibility of certain drugs in pregnancy.

### Future research agenda

The limitation of current evidence highlights the need for a national pregnancy registry for patients with rheumatic disease, as currently exists for women with epilepsy. All women with rheumatic disease who become pregnant would be eligible to register, whether or not they are on anti-rheumatic treatment. The prospective pregnancy outcome data would then be published to display information on outcomes such as miscarriage and congenital anomalies in patients treated with anti-rheumatic and other drug therapy. These data would also be used to answer specific questions where data is currently lacking. Data relating to the impact of paternal exposure to these drugs (both fertility and male-mediated teratogenicity), as well as breastfeeding exposure is particularly limited, and further research in these areas is urgently required.

### Mechanism for audit of the guideline

An audit pro forma to assess compliance with these guidelines is shown in Supplementary Data S1, available at *Rheumatology* online.

## Supplementary data

Supplementary data are available at *Rheumatology* online.

## Supplementary Material

keac552_Supplementary_Data

## Data Availability

All relevant data produced during the guideline development process are presented in the guideline or in the accompanying supplementary material.
