# Supplementary material for: British Society for Rheumatology guideline on prescribing drugs in pregnancy and breastfeeding: comorbidity medications used in rheumatology practice
Source: Rheumatology (Oxford). 2022 Nov 2;62(4):e89–e104. doi: 10.1093/rheumatology/keac552 (PMC10070063; doi:10.1093/rheumatology/keac552)
Supplement: keac552_Supplementary_Data [file keac552_supplementary_data.zip › rhe-22-1927-File008.docx]

**Supplementary Data S2. BSR Standards, Audit and Guidelines Working Group (SAGWG) members**

The members of SAGWG are as follows:

Ian Giles, Ed Roddy, Kate Armon, Lauren Astell, Caroline Cotton, Alan Davidson, Sarah Fordham, Claire Jones, Christopher Joyce, Anoop Kuttikat, Zoe McLaren, Karen Merrison, Devesh Mewar, Amanda Mootoo and Emma Williams.
