# Supplementary material for: British Society for Rheumatology guideline on prescribing drugs in pregnancy and breastfeeding: comorbidity medications used in rheumatology practice
Source: Rheumatology (Oxford). 2022 Nov 2;62(4):e89–e104. doi: 10.1093/rheumatology/keac552 (PMC10070063; doi:10.1093/rheumatology/keac552)
Supplement: keac552_Supplementary_Data [file keac552_supplementary_data.zip › 22-1927_Supplementary_Tables.docx]

**Supplementary Table S1. Details of search strategy**

| **No.** | Search terms |
| --- | --- |
| **1** | TI,AB(Pregnan*) |
| **2** | TI,AB(parent* n/5 expos* or Maternal n/5 expos* or mother* n/5 expos* or pregnan* n/5 expos* or prepregnancy n/5 expos* or antenatal n/5 expos* OR ante-natal n/5 expos* or trimester or trimester or conception OR pre-conception or conceiv*) |
| **3** | TI,AB(intrauterine expos* or in utero expos* or foetus expos* or foetal n/5 expos* or fetus n/5 expos* or fetal n/5 expos* or embryo n/5 expos* or embryonic n/5 expos* or pre-natal n/5 expos* or prenatal n/5 expos*) |
| **4** | TI,AB(lactat* or breast feeding or breastfeeding or postnatal expos*or post natal n/5 expos*or neonat* n/5 expos* newborn* n/5 expos* or bab* n/5 expos*) |
| **5** | TI,AB(Paternal n/6 expos* or father* n/6 expos* or paternal n/3 use or paternal n/3 usage or father* n/3 use or father* n/3 using or father* n/3 usage) |
| **6** | TI,AB(Live birth* or miscarriage* or stillbirth* OR Still birth*) |
| **7** | TI,AB(Ectopic pregnan* or fetal malformation* OR foetal malformation*) |
| **8** | TI,AB(congenital anomal* or congenital malformation*) |
| **9** | TI,AB(aborted n/3 pregnan* or abortion or congenital disorder* or birth anomal* or birth defect* or prematur*) |
| **10** | S1 OR S2 OR S3 OR S4 OR S5 OR S6 OR S7 OR S8 OR S9 |
| **11** | TI,AB(Analgesic*) |
| **12** | TI,AB(Paracetamol or Codeine or Morphine or tramadol or Gabapentin or Pregabalin) |
| **13** | TI,AB(Anticoagulant*) |
| **14** | TI,AB(Heparin or Warfarin or Rivaroxaban or Dabigatran or apixaban or edoxaban) |
| **15** | TI,AB(Clopidogrel) |
| **16** | TI,AB(Bisphosphonate*) |
| **17** | TI,AB(Alendronate or Etidronate or Risedronate or Pamidronate or Zoledronate or Denosumab) |
| **18** | TI,AB(NSAID* or anti-inflammator* or COX* n/3 inhibitor*) |
| **19** | TI,AB(Naproxen or Diclofenac or Ibuprofen or Indomethacin or Etodolac or Meloxicam or Celecoxib or etoricoxib or Colchicine or Dapsone) |
| **20** | TI,AB(Bosentan or Prostacyclin OR Sildenafil) |
| **21** | TI,AB(Amitriptyline or Nortriptyline or Duloxetine or Venlafaxine or Fluoxetine or Sertraline or paroxetine or aspirin or ACE inhibitor* or Angiotensin n/3 receptor or ARBs or Captopril or Imidapril or Enalapril or Lisinopril or Perindopril or Ramipril or Trandolapril or Cilazapril or Moexipril or Quinapril or Fosinopril or Losartan or Valsartan or Candesartan or Irbesartan or Eprosartan) |
| **22** | S11 OR S12 OR S13 OR S14 OR S15 OR S16 OR S17 OR S18 OR S19 OR S20 |
| **23** | EMB.EXACT("clinical pathway") or MJEMB.EXACT("clinical protocol") or EMB.EXACT.EXPLODE("consensus") or EMB.EXACT.EXPLODE("practice guideline") or EMB.EXACT("health care planning") |
| **24** | MESH.EXACT("Critical Pathways") or MESH.EXACT.EXPLODE("Clinical Protocols") or MESH.EXACT("Consensus") or MESH.EXACT.EXPLODE("Consensus Development Conference") or MESH.EXACT.EXPLODE("Guideline") or MESH.EXACT("Health Planning Guidelines") |
| **25** | TI,AB(position statement* or policy statement* or practice parameter* or best practice*) |
| **26** | TI,AB(standards or guideline or guidelines) |
| **27** | TI,AB(practice n/2 guideline* or treatment* n/2 guideline* or clinical n/2 guideline*) |
| **28** | TI(CPG or CPGs) |
| **29** | TI(consensus*) |
| **30** | TI,AB(critical n/2 path* or clinical n/2 path* or practice n/2 path* critical n/2 protocol* or clinical n/2 protocol* or practice n/2 protocol*) |
| **31** | TI,AB(recommendat*) |
| **32** | TI,AB(standard n/2 care or path n/2 care or paths n/2 care or pathway n/2 care or pathways n/2 care or map n/2 care or maps n/2 care or plan n/2 care or plans n/2 care) |
| **33** | TI,AB(screening n/2 algorithm* or examination n/2 algorithm*  or test n/2 algorithm*  or tested n/2 algorithm*  or testing n/2 algorithm*  or assessment* n/2 algorithm*  or diagnosis n/2 algorithm*  or diagnoses n/2 algorithm*  or diagnosed n/2 algorithm*  or diagnosing n/2 algorithm*) |
| **34** | TI,AB(pharmacotherap* n/2 algorithm*  or therap* n/2 algorithm*  or treatment* n/2 algorithm* or intervention* n/2 algorithm*) |
| **35** | S23 OR S24 OR S25 OR S26 OR S27 OR S28 OR S29 OR S30 OR S31 OR S32 OR S33 OR S34 |
| **36** | S10 AND S22 AND S35 |
| **37** | S5 AND S21 AND S35 |
| **38** | TI,AB(systematic OR state-of-the-art OR scoping OR literature OR umbrella) |
| **39** | TI,AB(review* OR overview* OR assessment) |
| **40** | S38 n/2 S39 |
| **41** | TI,AB("review* of reviews" OR meta-analy* OR metaanaly* or systematic n/2 assess* OR evidence n/2 assess* OR "research evidence" OR metasynthe* OR meta-synthe*) |
| **42** | MESH.EXACT("Systematic Reviews as Topic") OR MESH.EXACT("Meta-Analysis") OR MESH.EXACT.EXPLODE("Meta-Analysis as Topic") OR MESH.EXACT("Systematic Review") |
| **43** | EMB.EXACT("systematic review (topic)") OR EMB.EXACT.EXPLODE("meta analysis") or EMB.EXACT("systematic review") OR EMB.EXACT("meta analysis (topic)") |
| **44** | S40 OR S41 OR S42 OR S43 |
| **45** | S10 AND S22 AND S44 |
| **46** | S5 AND S21 AND S44 |
| **47** | S36 OR S37 OR S45 OR S46 |
| **48** | S47 AND YR(>=2013) |
|  |  |
|  |  |
|  |  |
|  | COHORT SEARCH |
| **1** | TI,AB(Pregnan* OR childbirth) |
| **2** | TI,AB(parent* n/5 expos* or Maternal n/5 expos* or mother* n/5 expos* or pregnan* n/5 expos* or prepregnancy n/5 expos* or antenatal n/5 expos* OR ante-natal n/5 expos* or trimester or trimester or conception OR pre-conception or conceiv*) |
| **3** | TI,AB(intrauterine expos* or in utero expos* or foetus expos* or foetal n/5 expos* or fetus n/5 expos* or fetal n/5 expos* or embryo n/5 expos* or embryonic n/5 expos* or pre-natal n/5 expos* or prenatal n/5 expos*) |
| **4** | TI,AB(lactat* or breast feeding or breastfeeding or postnatal expos*or post natal n/5 expos*or neonat* n/5 expos* OR newborn* n/5 expos* or bab* n/5 expos*) |
| **5** | TI,AB(Paternal n/6 expos* or father* n/6 expos* or paternal n/3 use or paternal n/3 usage or father* n/3 use or father* n/3 using or father* n/3 usage) |
| **6** | TI,AB(Live birth* or miscarriage* or stillbirth* OR Still birth*) |
| **7** | TI,AB(Ectopic pregnan* or fetal malformation* OR foetal malformation*) |
| **8** | TI,AB(congenital anomal* or congenital malformation*) |
| **9** | TI,AB(aborted n/3 pregnan* or abortion or congenital disorder* or birth anomal* or birth defect* or prematur*) |
| **10** | S1 OR S2 OR S3 OR S4 OR S5 OR S6 OR S7 OR S8 OR S9 |
| **11** | TI,AB(Paternal or father*) |
| **12** | S1 and S11 |
| **13** | S12 or S5 |
| **14** | TI,AB(Etidronate or Nortriptyline) |
| **15** | TI,AB(ACE inhibitor* or Angiotensin n/3 receptor or ARBs or Captopril or Imidapril or Enalapril or Lisinopril or Perindopril or Ramipril or Trandolapril or Cilazapril or Moexipril or Quinapril or Fosinopril or Losartan or Valsartan or Candesartan or Irbesartan or Eprosartan) |
| **16** | S10 AND S14 |
| **17** | S13 AND S15 |
| **18** | S16 OR S17 |
| **19** | S18 AND YR(>=2013) |
| Abbreviations:  AB – abstract; TI – title, | |

The article search was conducted using the above outlined search terms in Mesh terms and truncated terms. Description of search 1: The search was built around search terms describing pregnancy morbidity (search 1: line 1 – 9) and was combined in line 10. Relevant drugs were added in line 11 – 22). In line 23 -43 the type of article was identified. In line 44 – 48 the individual search blocks were combined and a time filter for studies published after 2013 was added.

Additional drugs were chosen for a cohort search (search 2). The search was built around search terms describing pregnancy morbidity (search 1: line 1 – 9), and were combined in line 10. Relevant drugs were added in line 14 – 15. The individual search blocks were combined and a filter for studies published after 2013 was added.

**Supplementary Table S2. Evidence supporting generic recommendations**

| **Generic recommendations** | **GRADE** | **SOA** | **References** |
| --- | --- | --- | --- |
| 1. Pre-conception counselling should be addressed by all healthcare professionals, with referral to professionals with relevant experience as appropriate, to optimise all therapy, including non-pharmacological options for chronic pain management during pregnancy. | 1A | 99.5 | 4, 5, 7, 8 |
| 2. The risks and benefits of drug treatment to mother and fetus should be discussed and clearly documented by all healthcare professionals involved in the patients care. | 1A | 99.0 | 4, 5, 7, 8, 9 |
| 3. The cause of pain and other symptoms should be assessed and managed appropriately. | 1B | 98.5 | 4, 5, 7 |
| 4. The requirement for analgesia should be assessed and minimum effective dose prescribed and titrated according to response. | 1B | 100 | 11, 14 |
| 5. Tricyclic antidepressants are preferred over other antidepressant medications to manage chronic pain. | 1B | 98.1 | 11, 30 |
| 6. Cessation of anti-depressant therapy that is being used as chronic pain medication in the post-natal period is not recommended, due to the risk of adverse impact on mood . | 1C | 96.0 | 11, 30 |
| 7. Low dose aspirin (≤150mg/day) is recommended in all patients at high risk for pre-eclampsia. | 1A | 99.5 | 40, 42 |
| 8. Low molecular weight heparin is the preferred anticoagulant. | 1A | 100 | 41, 51 |
| 9. Nifedipine is the preferred vasodilator. | 1B | 98.5 | 13, 18, 20, 40 |
| 10. Paternal drug exposure may reduce male fertility but has not been associated with adverse fetal development or pregnancy outcome. Although evidence is weak, we recommend that men are reassured about the safety of fathering a pregnancy whilst taking medicines to manage comorbidities as described in this guideline | 1C | 98.0 | 2, 70-77 |

Abbreviations: GRADE, Grading of Recommendations, Assessment, Development and Evaluations; SOA, strength of agreement.

**Supplementary Table S3. Evidence supporting recommendations on prescribing analgesics**

| **Recommendations for Paracetamol in pregnancy and breastfeeding** | **GRADE** | **SOA** | **References** |
| --- | --- | --- | --- |
| i) Paracetamol is the analgesic of choice and compatible peri-conception and throughout pregnancy. | 1B | 99.0 | 16 - 20 |
| ii) LactMed describes paracetamol as a good choice for analgesia and fever reduction in breastfeeding mothers. | 2C | 99.5 | 16 |
|  |  |  |  |
| **Recommendations for Codeine in pregnancy and breastfeeding** |  |  |  |
| i) Codeine is compatible peri-conception and throughout pregnancy, although long-term use should be avoided. There is no consistent evidence to recommend a dose reduction pre-delivery but neonatologists should be aware of maternal use. | 1B | 97.8 | 16, 17, 18, 21, 22, 23 |
| ii) Caution is advised with use of codeine in breast-feeding, due to the risk of CNS depression resulting from unpredictable metabolism of codeine to morphine. | 1C | 98.0 | 20,24,25,26 |
|  |  |  |  |
| **Recommendations for Tramadol in pregnancy and breastfeeding** |  |  |  |
| i) Avoid tramadol peri-conception and in first trimester and only consider in second/third trimester if no alternative analgesia. | 2B | 97.8 | 16, 18, 27, 28 |
| ii)  Based on limited data tramadol may be compatible with short-term use in breastfeeding. | 2C | 94.8 | 19, 20, 29 |

Abbreviations: CNS – central nervous system; GRADE, Grading of Recommendations, Assessment, Development and Evaluations; SOA, strength of agreement.

**Supplementary Table S4. Evidence supporting recommendations for the use of chronic pain medications**

| **Recommendations for Amitriptyline in pregnancy and breastfeeding** | **GRADE** | **SOA** | **References** |
| --- | --- | --- | --- |
| i) Amitriptyline is compatible with pregnancy. There is no evidence of adverse effect on IQ or developmental outcomes. | 1C | 100 | 16, 18, 30 |
| ii) Since very little amitriptyline is found in breast milk with antidepressant doses and it is used at lower doses for chronic pain, it is unlikely to cause adverse effects in breastfed infants. | 1C | 100 | 20 |
| **Recommendations for Gabapentin and Pregabalin in pregnancy and breastfeeding** |  |  |  |
| i) Gabapentin at lowest effective dose may be considered in pregnancy with folic acid supplementation if no alternative analgesic suitable grade. | 1B | 95.0 | 16, 18, 31 |
| ii) Gabapentin may be considered in breastfeeding if no alternative analgesic is suitable. | 2C | 96.0 | 20 |
| iii)  Pregabalin may be considered in pregnancy (with folic acid supplementation) and during breastfeeding | 2C | 95.3 | 18, 19, 20, 32, 33, 34, 35 |
| **Recommendations for SNRIs in pregnancy and breast-feeding** |  |  |  |
| i) Venlafaxine is compatible at conception and throughout pregnancy. There may be an increased risk of neonatal abstinence syndrome / short-term behavioural effects, but larger studies are needed to evaluate this finding. | 2C | 95.8 | 16, 18, 30, 33 |
| ii) Duloxetine may be considered in pregnancy and breastfeeding but there are less data than for venlafaxine. | 2C | 95.3 | 20, 30 |
| iii) Venlafaxine and duloxetine may be considered in breastfeeding if there is no alternative chronic pain medication. | 2C | 95.8 | 20 |
| **Recommendations for SSRIs in pregnancy and breast-feeding** |  |  |  |
| i) Fluoxetine, paroxetine and sertraline are compatible with pregnancy. | 1B | 98.8 | 16, 30, 35, 37, 38, 39 |
| ii) Based on limited evidence SSRIs are compatible with breastfeeding. | 2C | 98.3 | 20 |

Abbreviations: GRADE, GRADE, Grading of Recommendations, Assessment, Development and Evaluations; IQ – intelligence quotient; SNRI - Serotonin-norepinephrine reuptake inhibitors; SSRI - Selective serotonin reuptake inhibitors; SOA, strength of agreement.

**Supplementary Table S5. Evidence supporting recommendations on use of anti-inflammatory drugs**

| **Recommendations for NSAIDs, COX-2 inhibitors in pregnancy and breastfeeding** | **GRADE** | **SOA** | **References** |
| --- | --- | --- | --- |
| i) Discordant findings from retrospective, large studies with population controls on the use of non-selective NSAIDs in the first trimester of pregnancy raise the possibility of a low risk of miscarriage and malformation. Therefore, these drugs should only be used intermittently in the first trimester of pregnancy. | 1B | 97.3 | 16, 18, 40-44 |
| ii) Intermittend rather than regular use of all non-selective NSAID except LDA is recommended throughout pregnancy and weaned from end of second trimester (26 weeks) to stop by gestational week 30 to avoid premature closure of the ductus arteriosus | 1B | 98.0 | 16,45 |
| iii) At present there are limited data on selective cyclooxygenase-2 inhibitors; they should therefore be avoided during pregnancy. | 2C | 98.5 | 16,43 |
| iv)  Non-selective NSAIDs (especially Ibuprofen) are compatible with breastfeeding. | 1C | 98.8 | 20 |
|  |  |  |  |
| **Recommendations for colchicine and dapsone in pregnancy and breastfeeding** |  |  |  |
| i) Colchicine therapy may be considered during pregnancy. | 1B | 99.5 | 18, 46, 47 |
| ii) Dapsone may be used in pregnancy. | 2C | 95.0 | 48-50 |
| iii) Colchicine may be used in breastfeeding. | 2C | 98.3 | 20, 47 |
| iv) Dapsone may be used in breastfeeding and due to the risk of haemolytic anaemia it is advised to monitor the infant for signs of hemolysis, especially in newborn or premature breastfed infants. | 2C | 90.7 | 20 |

Abbreviations: CNS, central nervous system; COX-2, cyclooxygenase-2; GRADE, Grading of Recommendations, Assessment, Development and Evaluations; LDA, low dose aspirin; NICE, National Institute for Health and Care Excellence; NSAID, non-steroidal anti-inflammatory drugs;

SOA, strength of agreement.

**Supplementary Table S6. Evidence supporting recommendations on use of aspirin, clopidogrel and anticoagulation**

| **Recommendations for low dose aspirin and clopidogrel in pregnancy and breastfeeding** | **GRADE** | **SOA** | **References** |
| --- | --- | --- | --- |
| i) LDA may be continued throughout pregnancy and NICE guidelines (2019) for hypertension in pregnancy advises treatment with LDA (for prophylaxis of pre-eclampsia) until delivery. | 1B | 99.0 | 18, 40-42 |
| ii) LDA is compatible with breastfeeding. | 2C | 99.8 | 20, 39 |
| iii) There is limited data on clopidogrel but it may be considered where alternative drugs are not suitable in pregnancy and breastfeeding. | 2C | 96.3 | 18, 40, 44 |
|  |  |  |  |
| **Recommendations for anticoagulants in pregnancy and breastfeeding** |  |  |  |
| 1. LMWH is compatible throughout pregnancy. | 1A | 100 | 1,2, 18, 41, 51 |
| ii) LMWH is compatible with breastfeeding. | 1C | 100 | 20 |
| iii)  The use of warfarin in pregnancy is associated with increased fetal risk throughout pregnancy and has limited indications therefore should only be considered in exceptional circumstances. | 1B | 98.8 | 18, 53,54 |
| iv)  Warfarin is compatible with breastfeeding. | 1A | 100 | 20 |
| v) DOACs cannot be recommended in pregnancy. | 1C | 97.9 | –,55,56,57 |
| vi) Rivaroxaban may be considered in breast feeding. | 2C | 95.3 | 20, |
| vii) Other DOACs are not recommended in breastfeeding due to lack of human data and concerns from animal studies. | 1C | 97.4 | 20 |
| viii) Fondaparinux may be considered in pregnancy and breastfeeding if there is an allergy or adverse response to LMWH. | 2C | 95.5 | 20, 58 |

Abbreviations: DOACs, direct oral anticoagulants; GRADE, Grading of Recommendations, Assessment, Development and Evaluations; LDA, low dose aspirin; LMWH, low molecular weight heparin; NICE, National Institute for Health and Care Excellence; SOA, strength of agreement.

**Supplementary Table S7. Evidence supporting recommendations on use of bisphosphonates and pulmonary vasodilators**

| **Recommendations for Bisphosphonates in pregnancy and breastfeeding** | **GRADE** | **SOA** | **References** |
| --- | --- | --- | --- |
| i) There is insufficient data upon which to recommend bisphosphonates in pregnancy or to advise a specific time for them to be stopped pre-conception. Given their biological half-life in bone of up to 10 years and no evidence of harm from limited reports of their use in pregnancy, a pragmatic recommendation is that they should be stopped 3 months in advance of pregnancy. | 2C | 96.8 | 18, 59,60 |
| ii) There are no data on which to base a recommendation for the use of bisphosphonates during breastfeeding. | 2C | 98.5 | 20 |
| **Recommendations for pulmonary vasodilators in pregnancy and breastfeeding** |  |  |  |
| i)      Established moderate to severe pulmonary hypertension (PHT) remains a contraindication to pregnancy. If pregnancy occurs the use of these pulmonary vasodilator drugs in pregnancy should be considered only as part of a multidisciplinary team assessment. | 1C | 99.5 | 2 |
| ii)     Limited evidence supports the use of prostacyclines to treat PHT during pregnancy. | 2C | 98.0 | 2, 18 |
| iii)    Limited evidence supports the use of sildenafil to treat PHT during pregnancy. | 2C | 98.0 | 18, 65 |
| iv)    Bosentan is teratogenic in animals and although there is no evidence of harm from human pregnancy the evidence is insufficient to recommend in pregnancy. | 1C | 98.8 | 2, 18, ,68, 69 |
| v)     There are no data relating to breastfeeding exposure to pulmonary vasodilators on which to base a recommendation. | 2C | 98.8 | 20, 64 |

Abbreviations: GRADE, Grading of Recommendations, Assessment, Development and Evaluations; PHT, pulmonary hypertension; SOA, strength of agreement.

**Supplementary Table S8. Evidence supporting recommendations on use of antihypertensives**

| **Recommendations for ACEI/ARB in pregnancy and breastfeeding** | **GRADE** | **SOA** | **References** |
| --- | --- | --- | --- |
| i) Angiotensin converting enzyme inhibitors (ACEI) and Angiotensin Receptor Blockers (ARBs) should be stopped as soon as possible when pregnancy is confirmed in the first trimester and if necessary an alternative antihypertensive compatible with pregnancy given. | 1A | 100 | 18, 39, –61-63 |
| ii) ACEI/ARB should be avoided in the second and third trimester but may be considered under specialist advice in certain circumstances. | 1C | 98.5 | 18, 39 |
| iii) Based on limited evidence enalapril is compatible with breastfeeding. | 2C | 98.5 | 18, 39, 64 |
|  |  |  |  |
| **Recommendations for Calcium channel blockers in pregnancy and breastfeeding** |  |  |  |
| i) Nifedipine is compatible with pregnancy with no direct evidence of harm at doses up to 90mg/day. | 1A | 99.0 | 18, 39 |
| ii) Nifedipine is compatible with breastfeeding. | 1B | 100 | 20 |
| iii)  Amlodipine can be considered in pregnancy and breastfeeding as there is no evidence of harm. | 1C | 97.9 | 20, 39 |

Abbreviations: ACEi, Angiotensin converting enzyme inhibitors; ARBs, Angiotensin Receptor Blockers; GRADE, Grading of Recommendations, Assessment, Development and Evaluations; SOA, strength of agreement.

**Supplementary Table S9. Evidence supporting recommendations on paternal prescribing**

| **Paternal recommendations** | **GRADE** | **SOA** | **References** |
| --- | --- | --- | --- |
| i)      Paracetamol is compatible with paternal exposure. | 1B | 98.5 | 2, 70, 72 |
| ii)     Amitriptyline, SNRIs and SSRIs are compatible with paternal exposure. | 1B | 98.5 | 2, 69 |
| iii)    Non-selective NSAIDs are compatible with paternal exposure. | 1C | 98.4 | 2, 73,,75 |
| iv)    Based on limited or no data and no association with adverse fetal development or pregnancy outcome, paternal exposure to all other drugs described in this guideline are unlikely to be harmful. | 2C | 97.3 | 2, 70 - 77 |

Abbreviations: ACEI, Angiotensin-converting enzyme inhibitors; ARB, Angiotensin II receptor blocker; CCB, calcium channel blocker; GRADE, Grading of Recommendations, Assessment, Development and Evaluations; LDA, low dose aspirin; NSAID, non-steroidal anti-inflammatory drugs; SNRI - Serotonin and norepinephrine reuptake inhibitors; SOA, strength of agreement; SSRI - Selective Serotonin Reuptake Inhibitor.
