# Supplementary material for: British Society for Rheumatology guideline on prescribing drugs in pregnancy and breastfeeding: comorbidity medications used in rheumatology practice
Source: Rheumatology (Oxford). 2022 Nov 2;62(4):e89–e104. doi: 10.1093/rheumatology/keac552 (PMC10070063; doi:10.1093/rheumatology/keac552)
Supplement: keac552_Supplementary_Data [file keac552_supplementary_data.zip › rhe-22-1927-File007.docx]

##### **Supplementary Data S1. Audit tool**

**Audit tool: Prescribing anti-rheumatic therapy in pregnancy and breastfeeding audit tool (female)**

Which rheumatic disease(s) is (are) being treated?

Was the patient asked about their wish to conceive prior to conception? Y/N

If available, has the patient been referred to specialist pregnancy clinic? Y/N

Has the patient been given pre-pregnancy counselling on drug therapy? Y/N

Has the patient been given advice on further reading on drug therapy in pregnancy/breastfeeding? Y/N

Was appropriate medication advice given pre-conception? Y/N

Was appropriate medication advice given during pregnancy? Y/N

Was appropriate medication advice given during breastfeeding (if applicable)? Y/N

If inappropriate advice was given regarding medication changes pre-conception, during pregnancy or breastfeeding, in what way (s) did the advice differ from these guidelines?

Was patient monitored through pregnancy for drug efficacy/toxicity? Y/N

Was patient reviewed ≤ 4 months post-partum for drug efficacy/toxicity? Y/N

Was the baby reviewed for drug toxicity effects? Y/N

**Optional additional questions (for research purposes):**

Was choice of medication altered by patient’s wish to conceive (give details)? Y/N

Which drugs were advised to be continued throughout conception, pregnancy and breastfeeding?

Which drugs were advised to be stopped or withheld (state stage of pregnancy to be stopped, and when to be restarted)

*e.g. Methotrexate – stop 3 months pre-conception, re-start after stopping breastfeeding*

Did patient follow medical advice with regards to medication changes pre and peri conception, during pregnancy, and during breastfeeding? Y/N

Which drugs did the patient actually take?

Pre-conception & peri-conception?

During pregnancy (each trimester)?

During breastfeeding?

What was the outcome of this pregnancy?

Live birth? Y/N

If Y, were any malformations noted (specify)? Y/N

Spontaneous miscarriage? Y/N

If Y, which trimester?

Elective termination? Y/N

If Y, was this due to:

Theoretical drug exposure concerns? Y/N

Malformations noted during antenatal care? Y/N

Other reason (specify)?

**Prescribing anti-rheumatic therapy in pregnancy and breastfeeding audit tool (male)**

Which rheumatic disease(s) is (are) being treated?

Was the patient asked about their wish to conceive prior to conception? Y/N

Has the patient been given pre-conception counselling on drug therapy? Y/N

Has the patient been given advice on further reading on drug therapy during conception/ pregnancy? Y/N

Was appropriate medication advice given pre-conception? Y/N

Was appropriate medication advice given during partner’s pregnancy? Y/N

If inappropriate advice was given regarding medication changes pre-conception or during partner’s pregnancy, in what way (s) did the advice differ from these guidelines?

Was the baby reviewed for drug toxicity effects? Y/N

**Optional additional questions (for research purposes):**

Was choice of medication altered by patient’s wish to conceive (give details)? Y/N

Which drugs were advised to be continued throughout conception and partner’s pregnancy?

Which drugs were advised to be stopped or withheld (state when to be stopped, and when to be restarted)

Did patient follow medical advice with regards to medication changes during conception, and during partner’s pregnancy? Y/N

What drugs did the patient actually take:

peri-conception?

during partner’s pregnancy?

What was the outcome of partner’s pregnancy?

Live birth? Y/N

If Y, were any malformations noted (specify)? Y/N

Spontaneous miscarriage? Y/N

If Y, which trimester?

Elective termination? Y/N

If Y, was this due to:

Theoretical drug exposure concerns? Y/N

Malformations noted during antenatal care? Y/N

Other reason (specify)?
